# Sda1, a Cys_2_-His_2_ Zinc Finger Transcription Factor, Is Involved in Polyol Metabolism and Fumonisin B_1_ Production in *Fusarium verticillioides*

**DOI:** 10.1371/journal.pone.0067656

**Published:** 2013-07-03

**Authors:** Martha Malapi-Wight, Jonathon Smith, Jacquelyn Campbell, Burton H. Bluhm, Won-Bo Shim

**Affiliations:** 1 Department of Plant Pathology and Microbiology, Texas A&M University, College Station, Texas, United States of America; 2 Department of Plant Pathology, University of Arkansas, Fayetteville, Arkansas, United States of America; 3 Bioenvironmental Sciences Program, Department of Plant Pathology and Microbiology, Texas A&M University, College Station, Texas, United States of America; University of Wisconsin - Madison, United States of America

## Abstract

The ubiquitous ascomycete *Fusarium verticillioides* causes ear rot and stalk rot of maize, both of which reduce grain quality and yield. Additionally, *F. verticillioides* produces the mycotoxin fumonisin B_1_ (FB_1_) during infection of maize kernels, and thus potentially compromises human and animal health. The current knowledge is fragmentary regarding the regulation of FB_1_ biosynthesis, particularly when considering interplay with environmental factors such as nutrient availability. In this study, *SDA1* of *F. verticillioides*, predicted to encode a Cys-2 His-2 zinc finger transcription factor, was shown to play a key role in catabolizing select carbon sources. Growth of the *SDA1* knock-out mutant (Δsda1) was completely inhibited when sorbitol was the sole carbon source and was severely impaired when exclusively provided mannitol or glycerol. Deletion of *SDA1* unexpectedly increased FB_1_ biosynthesis, but reduced arabitol and mannitol biosynthesis, as compared to the wild-type progenitor. *Trichoderma reesei ACE1,* a regulator of cellulase and xylanase expression, complemented the *F. verticillioides* Δsda1 mutant, which indicates that Ace1 and Sda1 are functional orthologs. Taken together, the data indicate that Sda1 is a transcriptional regulator of carbon metabolism and toxin production in *F. verticillioides*.

## Introduction

In nature, fungi utilize a broad range of carbon sources for anabolism and energy [1]. Extensive efforts have elucidated carbon metabolic pathways in *Saccharomyces cerevisiae* that are broadly conserved among yeast species [2]. Molecular mechanisms underlying sugar utilization have been studied extensively among yeasts, particularly regarding glucose sensing and metabolism [3]. *S. cerevisiae* and many other yeasts utilize mono and disaccharides through conserved metabolic networks such as glycolysis, the tricarboxylic acid cycle, and the pentose phosphate pathway [1]. However, *S. cerevisiae* does not possess some of the more complex sugar metabolic pathways found in filamentous fungi, such as for the biosynthesis of polyols (sugar alcohols).

Polyols are formed by the reduction of a carbonyl to a hydroxyl on a monosaccharide. Filamentous fungi produce a wide range of polyols, including mannitol, which is one of the most common polyols found in nature [4], [5]. In filamentous fungi, polyols play important roles in tolerance to environmental stress by maintaining positive turgor pressure in cells [6]. Additionally, diverse polyols, *e.g*., mannitol, glycerol, erythritol and arabitol, are involved in a wide range of cellular processes, including osmotic protection, carbohydrate storage, spore dispersal, mating, and quenching of reactive oxygen species [6]–[9]. In mammalian cells, polyol biosynthesis can be activated by the presence of high glucose levels [10], where glucose is reduced to sorbitol by aldose reductase (AR), and subsequently oxidized to fructose by sorbitol dehydrogenase (SDH). SDH belongs to the superfamily of medium-chain dehydrogenase/reductases and is expressed ubiquitously in all mammalian tissues [11]. However, a clear fungal ortholog of mammalian SDH is not known to exist [12].

The ascomycete *Fusarium verticillioides* (Sacc.) Nirenberg (teleomorph: *Gibberella moniliformis* Wineland) causes stalk and ear rots on maize worldwide [13], [14]. In addition to causing economic losses due to reduced yield, the fungus is directly associated with fumonisin contamination of infested grain. Fumonisins are polyketide-derived secondary metabolites structurally similar to sphinganine, and are known to disrupt sphingolipid biosynthetic pathways [15]. The fumonisin most commonly found in nature is fumonisin B_1_ (FB_1_), a carcinogen associated with various toxicoses in humans and animals [16]–[19] that is subject to governmental regulation in feed and foodstuff [20]. Fumonisin biosynthesis requires a cluster of contiguous and co-regulated *FUM* genes [22]. *FUM1*, which encodes a polyketide synthase, is required for the synthesis of the core polyketide backbone of the fumonisins [21], [22], and other *FUM* genes are predicted to catalyze various downstream steps in fumonisin biosynthesis. Additionally, *FUM21*, which encodes a Zn(II)_2_Cys_6_ transcription factor (TF), is present in the *FUM* gene cluster, and is required for the transcriptional activation of *FUM* genes and fumonisin biosynthesis [23], [24]. Moreover, environmental factors such as nutrient availability influence fumonisin biosynthesis, although the underlying molecular mechanisms are poorly understood [23], [25], [26]. For instance, Bluhm and Woloshuk [27] showed that amylopectin is a key stimulant that triggers elevated levels of FB_1_ when *F. verticillioides* colonizes corn kernels. Nitrogen availability also influences FB_1_ biosynthesis, and the TF AreA serves as the key regulator of nitrogen metabolism and fumonisin biosynthesis [28]. However, knowledge is fragmentary regarding signal transduction pathways regulating FB_1_ biosynthesis in response to environmental factors [29], [30].

TFs with zinc fingers have been reported in organisms ranging from bacteria to humans. Significantly, 2% of the human proteome contains this motif and TFs with zinc fingers are the most common type of TF found in the human genome [31]. Among fungi, members of the Cys-2 His-2 (C_2_H_2_) TF family are predicted to regulate pathogenicity, cell differentiation, carbon utilization, and development [32]. One of our research aims is to systematically study how C_2_H_2_ TFs regulate primary and secondary metabolism in the maize pathogen *F. verticillioides*. Here, we investigated the role of *SDA1* (sorbitol dehydrogenase activator 1) on carbon metabolism, asexual development, and fumonisin production. We also tested the functional conservation between *Trichoderma reesei* Ace1 and *F. verticillioides* Sda1. *T. ressei ACE1* has been studied in detail due to its role in regulating the expression of various enzymes required for carbon utilization. *T. reesei* is the industrial source of enzymes to break down plant biomass into simple sugars, and *ACE1* regulates the expression of important cellulase and xylanase genes [33]. Ace1 contains three C_2_H_2_ zinc fingers at the C-terminus and binds the promoter of cellulase gene *CBH1 in vitro* [34]. In addition, deletion of *ACE1* resulted on severely impaired growth on medium containing sorbitol as the carbon source [34]–[36].

## Materials and Methods

### Fungal Strain, Culture Media, and Growth Conditions

*F. verticillioides* wild-type strain 7600 (Fungal Genetics Stock Center, Kansas City, MO) was stored in 30% glycerol at –80°C. Conidia were produced by culturing on V8 juice agar (200 ml of V8 juice, 3 g of CaCO_3_, and 20 g of agar per liter) at 25°C for 7 days. For growth rate studies, the strains were inoculated on 100 ml of defined liquid (DL) medium (pH 5.9) [37], containing 2% (w/v) of glucose, sorbitol, fructose, xylose, xylan or cellulose as the carbon source, with constant shaking (100 rpm) for 6 days at 25°C. Cultures were harvested, dried at 100°C for 24 hours, and weighed for biomass quantification. To assess production of microconidia, strains (0.5 cm-diameter agar plugs) were inoculated on V8 agar plates or cracked-corn medium [37] and incubated at 25°C in a 14 h light/10 h dark photocycle for 7 or 14 days, respectively. Conidia were harvested with 5 ml of distillated water (V8 plates) or 10 ml of 50% acetonitrile (cracked corn), and were counted with a haemocytometer.

### Nucleic Acid Manipulation and Quantitative Real Time RT- PCR (qRT-PCR)

*F. verticillioides* genomic DNA was extracted as previously described [37]. PCR primers used in this study are listed in Table S1. Southern analyses were performed following standard protocols [38], and the probes were ^32^P-labelled with the Prime-It II Random Primer Labeling kit (Stratagene, La Jolla, CA, USA). qRT-PCR analyses were performed in a Cepheid Smart Cycler system with a QuantiTect SYBR Green RT-PCR kit (Qiagen, Valencia, CA, USA) with 150 ng of RNA as template for each sample. The *F. verticillioides* β-tubulin gene *TUB2* (GeneBank accession no. U27303) was used for normalization.

### Gene-deletion, Complementation and Over Expression Constructs

The *SDA1* gene disruption cassette was designed for a split-marker recombination strategy [39]. DNA fragments corresponding to the 5′ (1,020 bp) and the 3′ (1,052 bp) flanking regions of *SDA1* were amplified with primer sets TF9-LF-F/TF9-LF-R and TF9-RF-F/TF9-RF-R, respectively. Then, partial *HYG* fragments designated HY (766 bp) and YG (924 bp) were amplified as previously described [40] with primer sets HYG/F-HY/R and YG/F-HYG/R, respectively. In a 1∶1 molar ratio, the 5′ flanking region-YG fragment and the 3′ flanking region-HY fragment were joined by PCR using primer sets TF-LF-F/YG-F and HY-R/TF9-RF-R, respectively. To complement the *SDA1* deletion mutant (Δsda1), the *SDA1* gene was amplified from the wild-type strain and co-transformed into the Δsda1 strain along with a geneticin resistance cassette. Specifically, plasmid pBS-G containing geneticin (G418)-resistance gene (*GEN*) was amplified with primers M13F and M13R, and the *SDA1* gene including 1 kb 5′ UTR and 1 kb 3′ UTR was amplified from genomic DNA with TF9-LF-F and TF9-LF-R primers using Expand Long Polymerase (Roche Molecular Biochemicals, IN, USA). After co-transforming *SDA1* and *GEN* into the *F. verticillioides* Δsda1 strain, colonies resistant to hygromycin and geneticin were isolated. A *F. verticillioides* strain constitutively expressing *ACE1* from *T. reesei* was created by transforming the Δsda1 strain with *ACE1* fused to the *Aspergillus nidulans gpdA* promoter (gpdAp), which was amplified with primers gpdA-F-Hyg tail and GPD-BamH-R (2,121 bp). The *ACE1* gene with 622-bp 3′ UTR was amplified with primers Ace1-BamH-F and Tr-Ace1-R, and gpdAp was fused to *ACE1* with restriction-site mediated technique by adding a BamH1 site to the GPD-BamH-R and Ace1-BamH-F Primers. Fragments were ligated using T4 DNA Ligase (New England Biolabs, MA, USA) and PCR amplified with primers GPDA-F-Hyg and Tr-Ace1-NesR. The resulting construct (gpdAp::*ACE1*) and *GEN* were co-transformed into the *F. verticillioides* strain Δsda1 and colonies resistant to hygromycin and geneticin were evaluated for phenotypic complementation.

### Fungal Transformation

Protoplasts of *F. verticillioides* wild-type 7600 and strain Δsda1 were produced and transformed as previously described [37] with minor modifications. Mureinase (2 mg/ml) was replaced with Driselase (5 mg/ml) (Sigma, St. Louis, MO) in the protoplast enzyme solution. Hygromycin- and geneticin-resistant transformants were selected on regeneration agar medium, containing either 150 µg/mL of hygromycin or geneticin as needed.

### FB_1_ Analysis

FB_1_ was measured as described by Shim and Woloshuk [41] with minor modifications. Three biological replicates of each fungal strain were grown on cracked corn (1.2 g dry weight medium for 14 days at 25°C). FB_1_ was extracted with acetonitrile:water (1∶1 v/v) for 24 hrs. The extracts were passed through equilibrated PrepSep SPE C18 columns (Fisher Scientific, Pittsburgh, PA, USA). FB_1_ samples were analyzed on a Shimadzu LC-20AT HPLC system (Shimadzu Scientific Instruments, Inc., Kyoto, Japan) equipped with an analytical Zorbax ODS column (4.6×150 mm^2^) (Agilent Technologies, Santa Clara, CA, USA) and a Shimadzu RF-20A fluorescence detector. FB_1_ was detected based on retention time and quantified by comparing peak areas with FB_1_ standards (Sigma). FB_1_ biosynthesis was normalized to ergosterol levels in samples by calculating [FB_1_ ppm/ergosterol ppm]×100 [42].

### Metabolite Profiling by Gas Chromatography

Polyol profiles were determined as described by Kim et al. [42]. Briefly, metabolites from ground kernels were extracted with methanol (500 mg kernel tissue per 2ml methanol) containing phenyl-β-D-glucopyranoside as the internal standard for normalization. Then, a portion of the extract from maize (150 µl) was transferred to a 2-ml autosampler vial and dried under a stream of nitrogen at ambient temperature. To deriviatize the extracted metabolites and internal standard, 100 µl of trimethylsilylimidazole:trimethylchlorosilane (100∶1, v:v) was added to each vial, thoroughly mixed, and incubated at 37°C for 1 hr. Derivatized products were selectively partitioned into the organic phase after adding 100 µl of isooctane followed by 200 µl of H_2_O. The aqueous and organic phases were separated by centrifugation at 2,500 rpm (1,260 g) for 2 min. The organic phase was collected, and 1 µl was injected with a split ratio of 10∶1 onto a 30 m×0.25 mm i.d. DB-5 column with a 0.10 µm stationary phase (Phenomenex, Torrance, CA, USA). The column oven temperature was held at 120°C for 5 min, increased to 300°C at 4°C/min, and then held constant at 300°C for 15 min. The FID temperature was 340°C. Temperature programming and data acquisition was performed with a GC-210 gas chromatograph (Shimadzu) controlled with Shimadzu GCsolution (V. 2.30). Target metabolites were identified and quantified based on retention times and peak areas, respectively, of analytical standards. Metabolite concentrations were normalized to the internal standard peak area and average ergosterol content. Samples obtained from liquid culture were analyzed as described by Smith et al. [43]. The procedure was essentially the same as for samples from maize kernels with the exception that 1.0 ml of methanolic extract was processed for analysis and metabolite concentrations were normalized to the internal standard peak area and tissue dry weight.

### Sexual Crosses

*F. verticillioides* sexual crosses were performed as described by Sagaram et al. [26]. Briefly, all strains were grown on V8 agar plates for 7 days and the wild-type strain 7598 (genotype MAT1-2) was transferred to carrot agar plates and incubated for 7 days. Subsequently, conidia of the wild-type strain 7600 and Δsda1 (MAT1-1 genotype) were harvested and applied to carrot agar plates covered with strain 7598 mycelia. Crosses were maintained at 25°C, with a 10 h dark and 14 h light photocycle until perithecia and ascospores were observed and characterized.

## Results

### Identification and Molecular Characterization of *SDA1*, a C_2_H_2_ Transcription Factor in *F. verticillioides*

**Figure 1:**
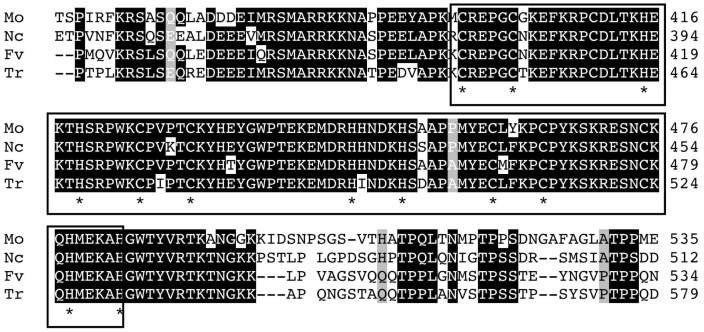
Amino-acid alignment of *F. verticillioides* (Fv) Sda1 and its comparison with predicted protein sequences of homologous proteins. Sda1 shares 52%, 50% and 65% identity with homologs in *Magnaporthe oryzae* (Mo) (GeneBank accession no. XP_003713522), *Neurospora crassa* (Nc) (GeneBank accession no. XP_963927) and *Trichoderma reesei* (Tr) (GeneBank accession no. Q9P8W3), respectively. The conserved residues were indicated by white letters on a black background. The regions corresponding to the three zinc fingers were indicated with boxes. Asterisks indicate the zinc coordinating Cys and His residues.

We identified a 3,555-bp gene with two introns (FVEG_01067.3) during our *in silico* screening of C_2_H_2_ TFs in the *F. verticillioides* genome [44]. The gene, designated *SDA1*, is located in supercontig 1 on chromosome 1, specifically from sequence 3,198,319 to 3,201,873. It is predicted to encode a 738-amino-acid polypeptide containing three C_2_H_2_ zinc finger domains at the C-terminus. BLAST analysis revealed a high level of similarity between the putative protein and a number of hypothetical, uncharacterized fungal proteins (Figure 1). However, the *Trichoderma reesei* Ace1 protein, which shares 65% identity and 76% similarity with Sda1 (Figure 1 and S1), has been further characterized due to its role in carbon metabolism. Ace1 negatively regulates expression of cellulases, hemycellulases and xylanases. In addition, a functional copy of *ACE1* is required for proper vegetative growth when D-sorbitol is the sole carbon source [45], [46].

**Figure 2:**
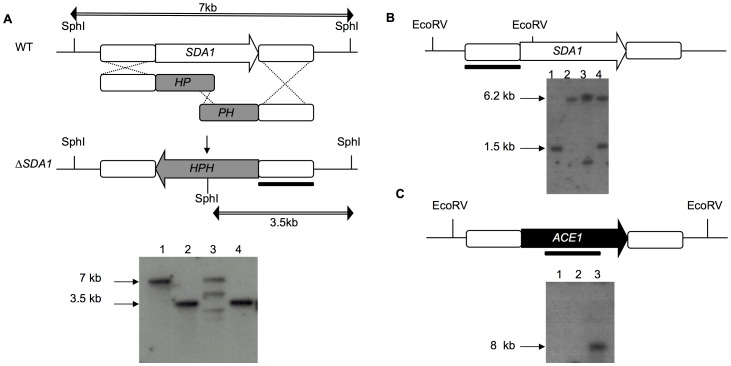
Schematic representation of the *SDA1* disruption and complementation strategies in *F. verticillioides*. (A) Target replacement of *SDA1* with the hygromycin phosphotransferase gene *(HPH)* by split marker technique through homologous recombination. Solid bar indicates DNA fragment used as the probe for Southern hybridization. Lanes: 1, wild-type; 2, Δsda1-7; 3, ectopic integration; 4, Δsda1-9. The wild-type strain produced a 7 kb band, and the Δsda1 knock out mutant produced a 3.5 kb band. (B-C) Schematic representations of constructs used to complement the Δsda1 strain. (B) *F. verticillioides SDA1* wild-type copy construct. Lanes 1, wild-type; 2, Δsda1; 3 and 4, Δsda1-complements (sdaC). The wild-type strain produced a 1.5 kb band and the Δsda1 knock out mutant produced a 6.2 kb band. The complemented sdaC strains produced a 6.2 kb and a random size band due to ectopic integration of the complementation construct. (C) *T. reesei ACE1* coding region fused to *A. nidulans* GPD promoter construct. Lanes 1, wild-type; 2, Δsda1; 3, Δsda-complement (sdaT). The complemented sdaT strain produced a single a random size band due to ectopic integration of the complementation construct.

To study *SDA1* in *F. verticillioides*, we generated a gene-deletion mutant with a split marker homologous recombination approach (Figure 2A) [39]. PCR and Southern analysis (Figure 2A) identified two strains from 30 hygromycin-resistant transformants in which the *SDA1* locus was successfully targeted. Subsequently, we complemented the gene-deletion mutant (Δsda1) with the wild-type *SDA1* gene along with its native promoter and terminator. We then used PCR and Southern analysis to identify a strain (sdaC) containing a single copy of the complementation construct (Figure 2B).

### *SDA1* Deletion Impaired Conidiation and FB_1_ Biosynthesis, but not Sexual Reproduction

**Figure 3:**
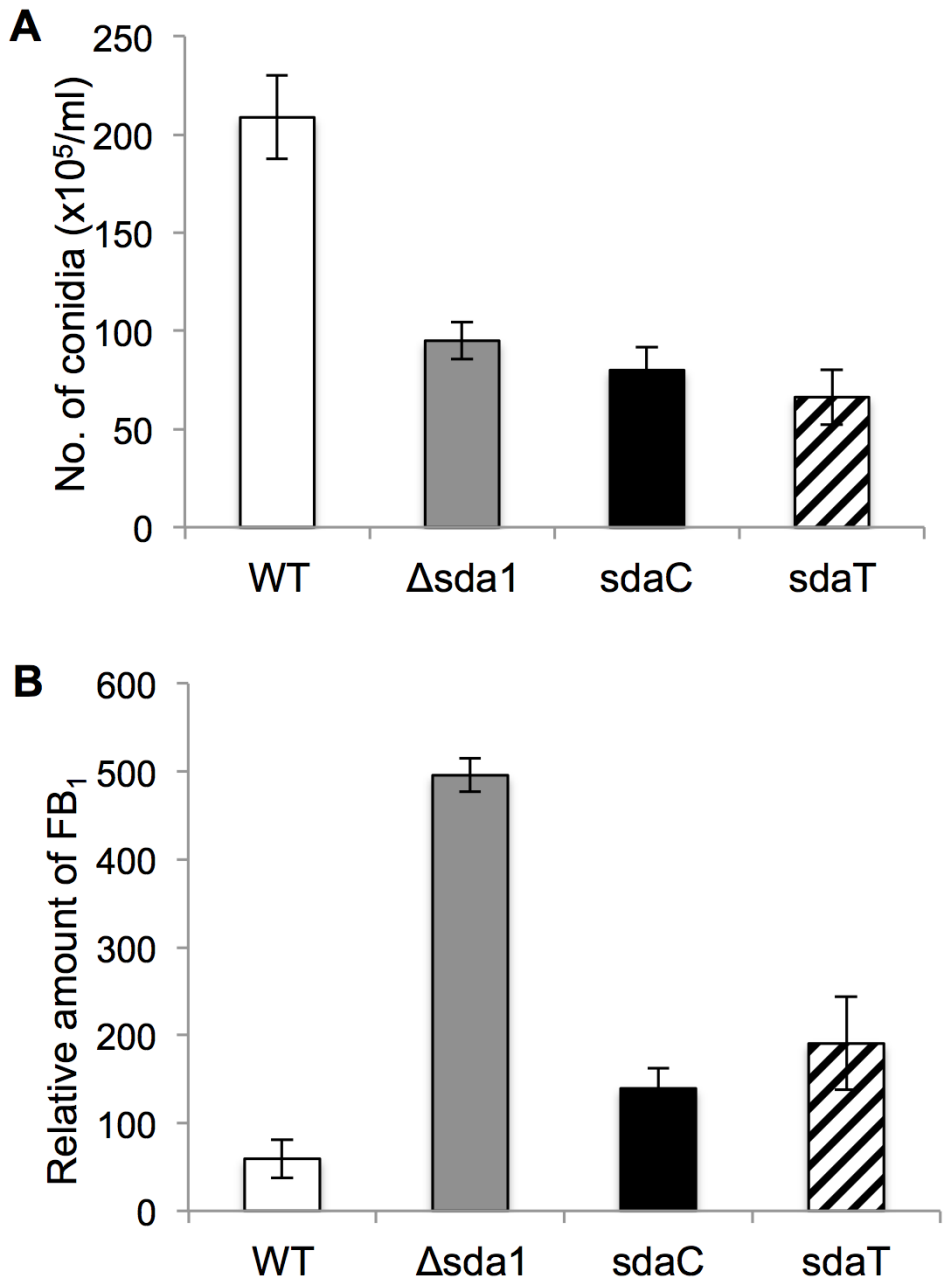
Quantification of microconidia and fumonisin B_1_ (FB_1_) production in *F. verticillioides* strains. Wild-type (WT), Δsda1, sdaC, and sdaT strains were point inoculated with an agar plug (0.5 cm in diameter) on nonviable autoclaved maize kernels and incubated for 14 days at 25°C under a 14-h light/10-h dark cycle. (A) Microconidia were harvested and quantified with a haemocytometer. (B) FB_1_ production was quantified by high-performance liquid chromatography (HPLC) analysis. FB_1_ biosynthesis was normalized to growth with ergosterol contents. All values represent the means of three biological replications with standard errors shown as error bars, and two independent experiments showing similar results.

Functional analyses of C_2_H_2_ TFs in other fungal species indicated that Sda1 might be involved in the regulation of asexual development [47], [48]. To test this, fungal strains were inoculated on V8 agar and autoclaved corn kernels. V8 agar plates were incubated for 7 days at 25**°**C, and a 20% decrease in microconidia production was observed in the Δsda1 strain compared to the wild type (data not shown). The reduction in conidiation was substantially more pronounced when strains were incubated on corn kernels, as the Δsda1 strain produced 50% less microconidia compared to the wild-type strain (*P*<0.01) (Figure 3A). Inexplicably, conidiation was not fully restored in the sdaC strain as compared to the wild type (Figure 3A). The expression of *SDA1* in the sdaC strain was measured in DL medium amended with glucose and sorbitol as the carbon source (Table S2). However, the levels of *SDA1* expression in sdaC were significantly higher when compared to the wild-type strain in both media tested, suggesting that the partial complementation in the sdaC strain is not due to the defect in *SDA1* expression. The difference could be resulting from non-target effects that can occur during ectopic integration of the complementation construct, as previously reported in other *Fusarium* studies [49]. Sda1 was not associated with sexual reproduction in *F. verticillioides*. When the mutant strains were crossed with the wild-type strain with the opposite mating type on carrot agar, they successfully produced perithecia with viable ascospores (data not shown). In addition, the strains were inoculated on germinating maize kernels to assay seedling and kernel rot; no differences were observed (data not shown).

In filamentous fungi, there is often a positive correlation between asexual reproduction and secondary metabolism [50], [51]. Earlier studies in *F. verticillioides* have supported this concept, showing that microconidia production and FB_1_ biosynthesis are associated in some cases [37]. Based on the reduced conidiation of the Δsda1 strain, we postulated that *SDA1* deletion negatively impacts FB_1_ biosynthesis. To test this hypothesis, maize kernels were inoculated with Δsda1 or the wild type, and fungal growth and FB_1_ levels were measured after 14 days. When FB_1_ production was normalized to fungal growth (Figure S2), the Δsda1 strain produced five-fold more FB_1_ than the wild-type strain (*P*<0.001) (Figure 3B). Therefore, we concluded that Sda1 is required for proper asexual development, but also serves as a negative regulator of FB_1_ biosynthesis.

### *SDA1* Deletion Impairs Polyol Utilization

**Figure 4:**
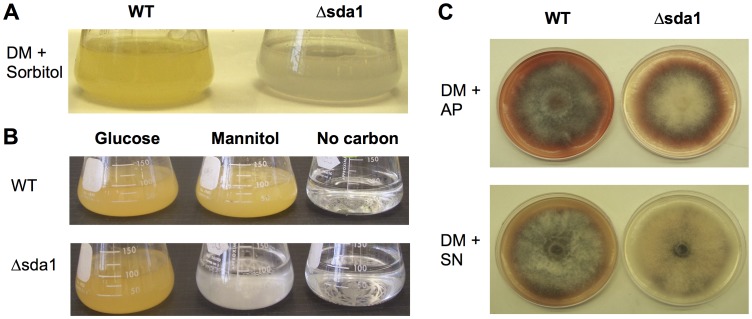
Growth rate and colony morphology of *F. verticillioides* wild-type (WT) and Δsda1 strains on various liquid and solid media. Strains were grown on defined liquid medium (DM) amended with 2% (A) sorbitol or (B) glucose, mannitol and no carbon. DM with 2% of glucose is the standard medium. Strains were inoculated in DM and incubated with constant shaking (100 rpm) for 6 days at 25°C. (C) Colony morphology of wild-type (WT) and Δsda1 strains on DM agar plates amended with ammonium phosphate (AP: NH_4_H_2_PO_4_) and sodium nitrate (SN: NaNO_3_). Strains were point inoculated with an agar plug and grown for 10 days at 25°C.

**Figure 5:**
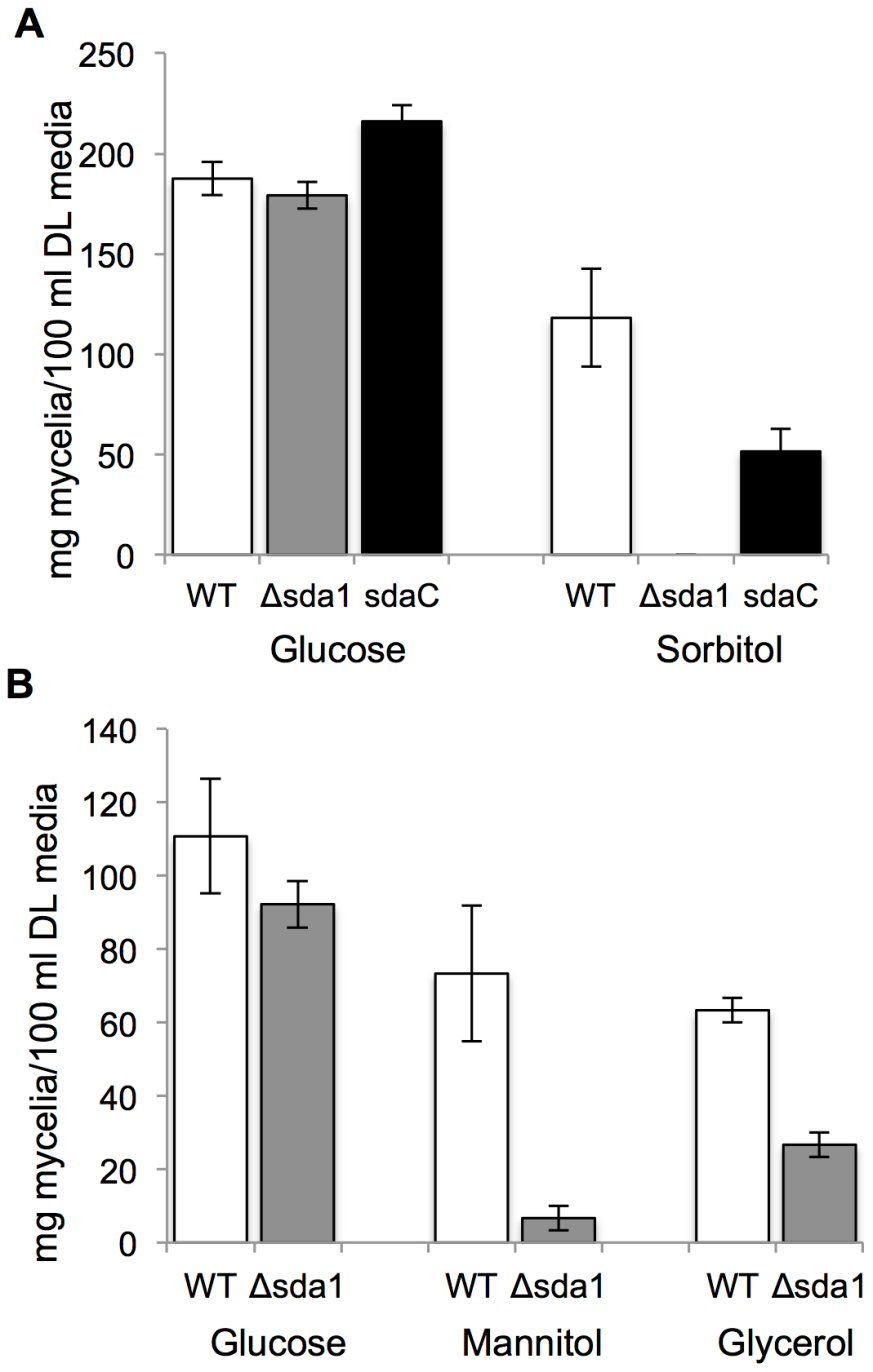
Biomass quantification and growth rate of *F. verticillioides* strains. Wild-type (WT), Δsda1, and sdaC strains were grown on DL media amended with 2% of (A) sorbitol, (B) mannitol and glycerol. DL media with 2% of glucose was used as control. The mycelia of each strain were harvested after 6 days of incubation in DL medium and dried at 100°C for 24 hrs. Results are the means of three and four biological replications with standard errors shown as error bars, and repeated at least twice.

Among fungi, a number of C_2_H_2_ TFs are important regulators of carbon metabolism [32]. For example, Ace1, a C_2_H_2_ TF in *T. reesei*, was identified as a positive and negative transcriptional regulator of various enzymes required for carbon utilization [34]. Notably, deletion of *ACE1* severely impaired growth on culture medium containing sorbitol as the sole carbon source [34]. In this context, the role of *SDA1* in carbon utilization was investigated. We cultured the Δsda1 and wild-type strains in defined liquid (DL) medium with sorbitol or glucose as the sole carbon source (Figure 4 and 5). D- sorbitol is a polyol that has the same linear structure as glucose, but the aldehyde group is replaced with a hydroxymethyl group. On DL medium with glucose as the sole carbon source, growth of the Δsda1 and wild-type strains were indistinguishable. However, in DL medium exclusively containing sorbitol as the carbon source, Δsda1 growth was drastically impaired (*P*<0.01) compared to the wild type or complemented strain sdaC (Figure 4A and 5A). Nitrogen source did not have a significant impact on the growth of mutant (Figure 4C).

To determine whether the Δsda1 growth defect resulted from an inability to utilize sorbitol or sorbitol toxicity, we inoculated the wild-type and Δsda1 strains on DL agar plates amended with 2% sorbitol. Radial growth of each strain was monitored at 3, 5 and 7 days-post inoculation; however, no significant difference in growth was observed (Figure S3). This indicated that sorbitol, or its derivatives, in the medium are not toxic to the mutant strain, but rather that sorbitol cannot be utilized by the mutant. Furthermore, the Δsda1 strain grew significantly less than the wild type in DL media containing mannitol (*P*<0.05) or glycerol (*P*<0.01) as the sole carbon source (Figure 4B and 5B). We did not observe any growth in both wild-type and mutant strains when cultured in DL with tergitol or without a carbon source (data not shown).

*T. reesei* Ace1 has been identified as a TF that mediates carbon catabolite repression of cellulases and xylanases when appropriate carbon sources are added [34], [46]. Aro et al. [34] showed that the Δace1 strain grew better on cellulose-based medium on which the expression of cellulases are needed for the fungus to grow, hence the name *ACE1* (*A*ctivator of *C*ellulase *E*xpression *1*). We inoculated our strains in DL medium supplemented with cellulose and harvested mycelia 6 days post-inoculation, when cellulases are presumed to be expressed actively [34]. However, we did not observe a significant difference in dry weight between the wild type and the Δsda1 strain (Figure S4). The experiments were repeated with xylose or xylan in DL, but we also did not observe a difference between strains under these conditions (Figure S4). These results demonstrated that *SDA1* is not involved in the “activation of cellulase” in *F. verticillioides*, and convinced us that *ACE1* (or *FvACE1*) is not a suitable name fore this gene.

### Deletion of *SDA1* Impairs Microconidia Germination in Sorbitol Medium

**Figure 6:**
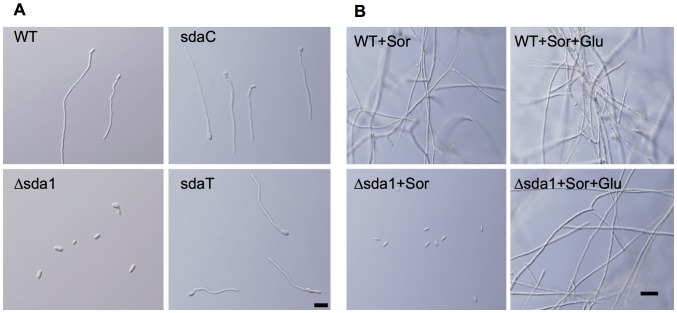
Germination and growth of *F. verticillioides* wild-type (WT), Δsda1, sdaC, and sdaT strains. A total of 10^5^ spores were inoculated and incubated in DL media with sorbitol at 25°C for (A) 16 h and (B) 40 h. (A) Note the lack of germination of the Δsda1 strain. (B) After 24 h of incubation in DL media with sorbitol, 2% w/v of glucose was added to the cultures. Note the germination of the Δsda1 strain in the presence of glucose vs. sorbitol. Scale bar = 5 µm.

In preliminary analyses, the mutant (Δsda1) strain had a pronounced growth defect on media containing sorbitol, which is consistent with the hypothesis that selected polyols induce dormancy in conidia when *SDA1* is disrupted. To test this, DL medium amended with sorbitol was inoculated with conidia of the Δsda1, sdaC, and wild-type strains. After 16 hours, germination of conidia from the sdaC and wild-type strains approached 100%, whereas Δsda1 conidia were significantly impaired in germination (Figure 6A). Significantly, when glucose was added to the culture to achieve 2% final concentration (w/v), Δsda1 conidia germinated and grew similar to the wild-type strain (Figure 6B). This result led us to hypothesize that Sda1 is an important transcriptional regulator for sorbitol catabolism in *F. verticillioides*.

### Sorbitol-induced Gene Expression

To determine if Sda1 regulates genes associated with sorbitol metabolism, we searched for putative sorbitol dehydrogenase (SDH) genes in *F. verticillioides*. Three predicted genes were identified as possible SDHs: *XDH1* (FVEG_03510.3), *SDH1* (FVEG_03564.3), and *SDH2* (FVEG_10437.3) (Table 1). The Xdh1 protein shares 54%, 84% and 98% amino acid identity to the sorbital/mannitol dehydrogenase (MtDH) of *Candida albicans* [52], *T. reesei* [30] and *Fusarium graminearum* [7], respectively. Trail and Xu [7] determined that *F. graminearum* MtDH showed enzymatic activity with sorbitol as the substrate, albeit only 21% when compared to using mannitol. Sdh1 shares 42% and 95% protein identity with *Homo sapiens* SDH and *F. graminearum* xylitol dehydrogenase (XDH), respectively. XDH can utilize sorbitol as a substrate, and is thought to be closely related to SDH [12]. Lastly, Sdh2 shares 45% identity at the protein level with the recently characterized SdhA in *A. niger* [53]. As anticipated, *SDA1* was up-regulated when *F. verticillioides* was grown in DL with sorbitol as the carbon source (Table 1). The qRT-PCR analyses indicated that *SDH1* expression was significantly increased (*P*<0.05) when the wild-type strain was grown on sorbitol versus glucose. However, no differences were observed in expression of *XDH1* or *SDH2.*

**Table 1:** Transcription levels of genes corresponding to *SDA1* and putative sorbitol dehydrogenases in DL medium containing either glucose or sorbitol as the sole source of carbona,c.

Gene	Gene expression in glucose^b^	Gene expression in sorbitol^b^
FVEG_01067.3 (*SDA1*)	1±0.16	4.09±0.4
FVEG_03510.3 (*XDH1*)	1±0.33	2.28±0.36
FVEG_03564.3 (*SDH1*)	1±0.38	2.22±0.38
FVEG_10437.3 (*SDH2*)	1±0.47	2.00±0.64

a Total RNA samples were prepared from *F. verticillioides* wild-type strain grown on DL media +2% w/v glucose or sorbitol. Mycelia were collected after 60 hours post inoculation. Real time quantitative reverse transcription (qRT)-PCR analysis of gene expression was performed with SYBR-Green as the fluorescent reporter. Gene expression was normalized to endogenous β-tubulin gene expression.

b The gene expression was calibrated using 2^−ΔΔCt^ method. Data represent the relative expression, where gene expression in glucose is standardized to 1.00± the standard error of dCT values (*n = 3*).

c Each value is the mean of 3–4 technical replicates from one biological experiment. A biological replication was performed with no statistically different results.

### Arabitol Accumulation is Decreased in Δsda1 Compared to Wild Type on Kernels

**Figure 7:**
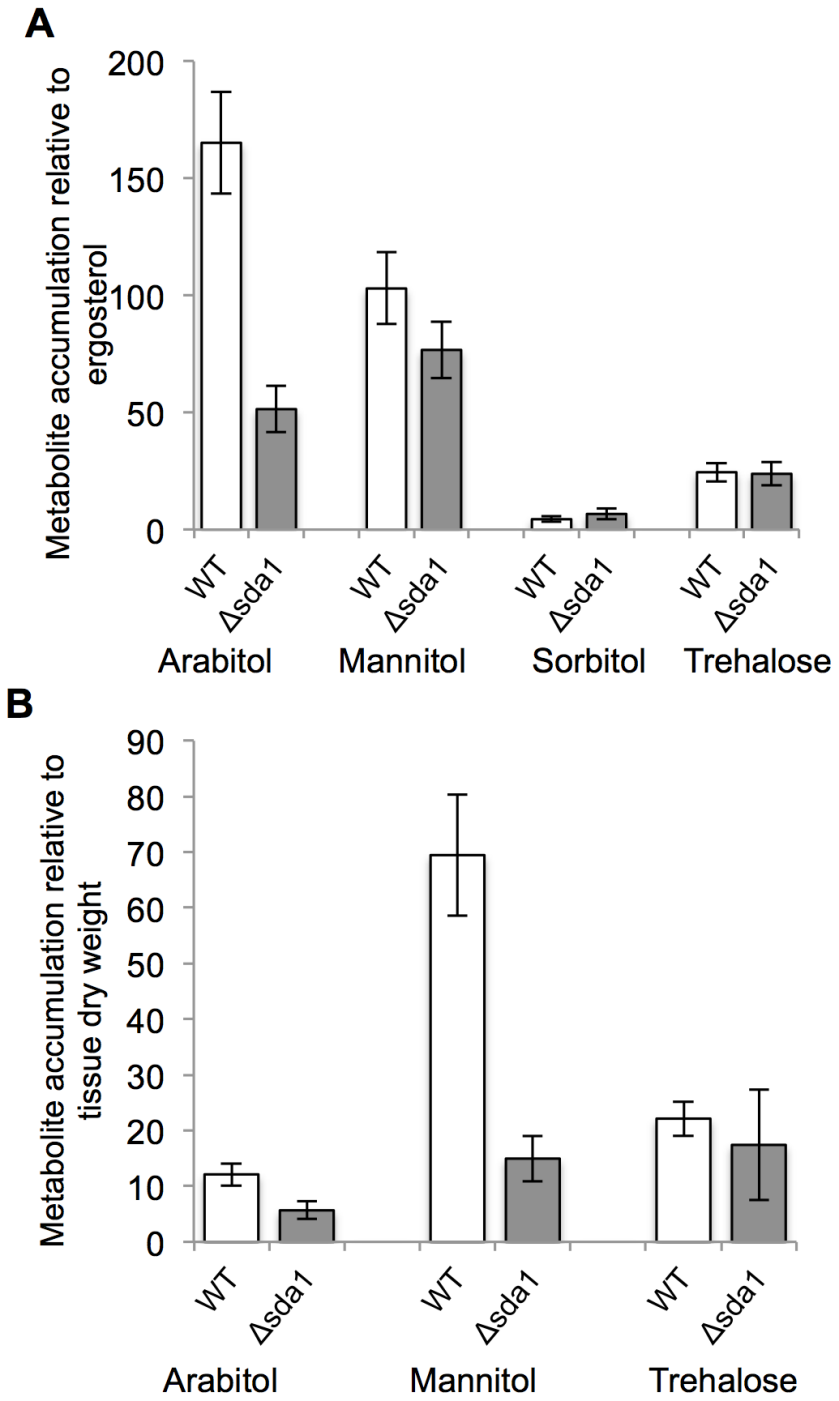
Accumulation of polyols and trehalose in maize kernels and liquid media. Accumulation of indicated metabolite in wild-type and Δsda1 grown for 7 days (A) on maize kernels and, (B) in liquid media containing glucose as the sole carbon source. Analyses were performed with at least four biological replicates. Results are the means of three and four biological replications with standard errors shown as error bars.

After observing that Δsda1 cannot grow on sorbitol, we performed a metabolic profiling experiment to determine the effects of *SDA1* deletion on carbohydrate metabolism in *F. verticillioides*. We inoculated fungal strains on corn kernels, and subsequently analyzed for a disaccharide (trehalose) and polyols (xylitol, arabitol, mannitol, and sorbitol). Xylitol was not detected in quantifiable concentrations in either the wild type or Δsda1 strains. The lack of xylitol accumulation in both strains suggests that either xylitol is not produced by *F. verticillioides* during colonization of corn kernels or is rapidly metabolized to downstream products. Quantifiable amounts of arabitol, mannitol, sorbitol, and trehalose were detected in kernels inoculated with both strains (Figure 7A and S5). However, significant differences in accumulation between wild type and Δsda1 were only detected for arabitol, with the wild-type strain accumulating approximately 3.2 fold more arabitol than Δsda1 (*P*<0.05) (Figure 7A). This decreased accumulation of arabitol in Δsda1 suggests that Sda1 contributes, either directly or indirectly, to arabitol biosynthesis in *F. verticillioides* during corn kernel colonization.

### Mannitol and Arabitol Accumulation is Decreased in Δsda1 Compared to Wild Type in Liquid Media

In addition to measuring carbohydrate accumulation in inoculated corn kernels, metabolic profiling was performed with wild type and Δsda1 strain in DL medium containing glucose as the sole carbon source. Similar to results obtained for kernels, quantifiable xylitol was not detected in either strain when grown in this medium. Sorbitol was also not detected in either strain, which contrasts with metabolic profiles from inoculated kernels (Figure 7B and S5). In DL with glucose, there was a significant difference in arabitol accumulation between the wild type and Δsda1, with the mutant accumulating approximately half as much arabitol as the wild type (*P*<0.05) (Figure 7B). This difference in arabitol accumulation is smaller compared to the difference observed in inoculated kernels, and it suggests that the role of Sda1 in arabitol metabolism is at least partially substrate dependent. Additionally, approximately 4.6-fold more mannitol was detected in the wild type than in Δsda1 when grown in DL with glucose (*P*<0.05) (Figure 7B). This reduction in mannitol accumulation in Δsda1 indicates that Sda1 also regulates mannitol metabolism in a substrate-dependent manner.

### *F. verticillioides* Sda1 and *T. reesei* Ace1 are Functional Orthologs

**Figure 8:**
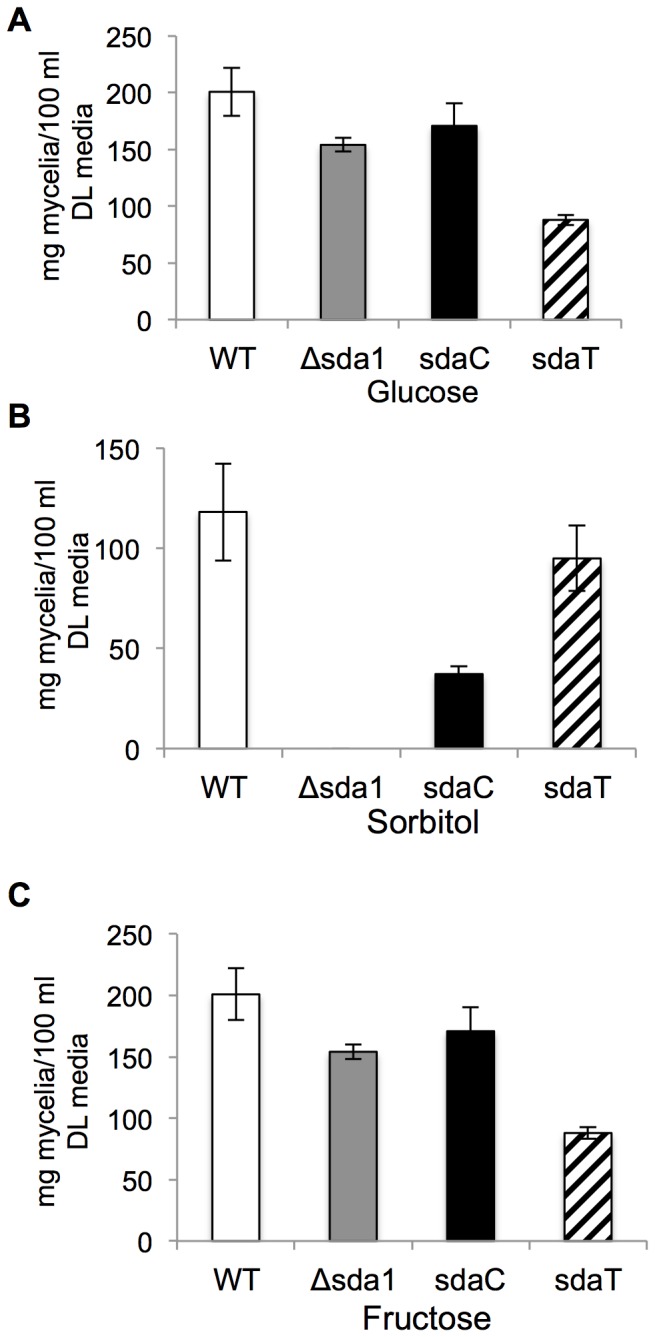
Biomass quantification of *F. verticillioides* strains. Wild-type (WT), Δsda1, sdaC, and sdaT strains were grown on DL media amended with 2% of glucose, sorbitol or fructose. The mycelia of each strain were harvested after 6 days of incubation in DL medium and dried at 100°C for 24 hrs. Results are the means of three and four biological replications with standard errors shown as error bars, and repeated at least twice.

We introduced *T. reesei ACE1* gene into the *F. verticillioides* Δsda1 strain in an effort to determine whether *ACE1* can functionally complement the *sda1* null mutation. Our initial approach was to transform with the complete *ACE1* coding region, which includes native promoter and terminator. However, because the publicly available *T. reesei* genome sequence is incomplete, we were not successful in amplifying the promoter region. Thus, we fused the *ACE1* gene to the *A. nidulans gpdA* promoter as an alternative approach (Figure 2C) and subsequently transformed the construct into the Δsda1 strain. The *A. nidulans gpdA* promoter has been shown to successfully drive constitutive gene expression in *F. verticillioides* [28]. One hundred transformants, resistant to both hygromycin and geneticin, were selected for further analyses by phenotypic observation, PCR and Southern blot (Figure 2C). Three transformants, sdaT-8, sdaT-43 and sdaT-44, contained the *T. reesei ACE1* gene and showed rescued, wild-type phenotypes. Further analysis of the sdaT-8 strain showed that complementation with *ACE1* restored growth on sorbitol (Figure 8), alleviated de-repression of FB_1_ biosynthesis (Figure 3B), and removed the inhibitory effect of sorbitol on germination of conidia (Figure 6A). However, conidiation was not restored in the sdaT strain to the wild-type level (Figure 3A), as was observed when the Δsda1 strain was complemented with the native *SDA1* of *F. verticillioide*s.

## Discussion

Polyols have a broad array of functions in filamentous fungi, particularly with regard to ameliorating environmental stresses such as osmotic, oxidative, and high temperature stresses [9], [54], [55]. In addition, polyols play a role in carbohydrate storage, spore dispersal, mating, and other aspects of growth and development [29]. Despite the importance of polyols, we have a very limited understanding of how higher fungi sense and utilize polyols and how the metabolic utilization of polyols influences fungal physiology. To the best of our knowledge, a report by Smith et al. [43] is the only study showing a correlation between environmental signals and polyol biosynthesis in *F. verticillioides*.

Carbon availability is another key environmental factor regulating FB_1_ biosynthesis in *F. verticillioides*. Bluhm and Woloshuck [27] showed that the presence of amylopectin during kernel colonization induces high levels of FB_1_ production. Likewise, elevated FB_1_ levels have been observed on mature kernels, possibly due to the higher starch concentration in the endosperm [56]. Regardless, signal transduction pathways regulating FB_1_ biosynthesis in response to carbon metabolites are not completely understood [42]. A limited number of zinc finger TFs are known to regulate the expression of *F. verticillioides FUM* genes, which directly influences FB_1_ biosynthesis [23], [28], [57], [58]. For instance, the C_2_H_2_ TF Pac1 is an ortholog of *A. nidulans* pacC, which is a transcriptional regulator of pH-responsive pathways. Flaherty et al. [57] showed that a *pac1* disruption mutant, when compared to the wild-type progenitor, produced higher levels of FB_1_ in acidic media. Moreover, the mutants produced FB_1_ in alkaline medium in contrast to the wild type. These results suggested that *F. verticillioides* Pac1 is a pH-regulatory TF, and may serve as a repressor of FB_1_ biosynthesis under alkaline conditions. Significantly, Son et al [59] recently reported that the Sda1 homolog GzC2H094 in *F. graminearum* plays a key role in sexual development, but not in mycotoxin production. However, this report is in contrast with our observations that the *F. verticillioides SDA1* deletion strain produced approximately 5-fold higher FB_1_ levels than the wild-type progenitor. Based on this result, we postulate that Sda1 is a negative regulator of FB_1_ production and that toxin regulation by this TF is not conserved among *Fusarium* species. The most direct mechanistic explanation is that Sda1 binds to the promoters of select *FUM* genes, thus blocking the recognition site for transcription and hindering FB_1_ production. However, additional experiments are needed to test this hypothesis.

The Δsda1 strain showed poor growth in the presence of mannitol and glycerol, and almost no growth when sorbitol was provided as the only carbon source. Our data are consistent with observations in *T. reesei*, in which deletion of Ace1, a putative orthologue of Sda1, caused a severe growth defect in the presence of sorbitol [34]. In eukaryotes, one metabolic pathway has been described for sorbitol catabolism, in which the NAD-dependent SDH oxidizes D-sorbitol to D-fructose [53]. SDHs share a high degree of homology at the amino-acid level with mannitol dehydrogenases (MtDHs), which suggests a functional conservation between these enzymes [7]. In *F. graminearum*, MtDH showed activity with mannitol as well as sorbitol [7]. Moreover, MtDH Lxr1 in *T. reesei* showed activity on every carbon source tested [30]. Our results, along with these published reports, suggest that dehydrogenase activities are not specific to a single substrate, but rather dependent upon the binding site of substrates [60]. The lack of growth by the Δsda1 strain in medium containing sorbitol as the exclusive carbon source indicates that Sda1 is required for sorbitol catabolism. Therefore, we postulate that Sda1 regulates transcriptional activation of SDH genes, which are necessary for the oxidation of sorbitol to fructose.

The amino-acid sequence of Sda1 shows a high degree of similarity to the C_2_H_2_ TF Ace1 from *T. reesei*. In this study *T. reesei ACE1*, under the control of the *A. nidulans gpdA* promoter, functionally complemented the Δsda1 strain by enabling growth in sorbitol medium and restoring wild-type levels of FB_1_ production on corn kernels. Deletion of the *ACE1* gene in *T. reesei* increased the expression of cellulases and xylanases under inducing conditions, and augmented growth in medium containing cellulose [34], [45], [46]. In addition, growth of the *ace1* deletion strain was severely impaired in the presence of sorbitol [34]. Ace1 contains three C_2_H_2_ zinc fingers at the C-terminus and it has been shown to bind *in vitro* to eight promoter sites of the major cellulose cellobiohydrolase I (*CBH1*) [45]. Based on these and other results, Ace1 is predicted to be a repressor of cellulase and xylanase expression. A mechanism explaining the down regulation of cellulose expression by Ace1 has been proposed; Ace1 may regulate, under inducing conditions, the balance between the levels of mRNA transcribed and the rate of enzyme translated/secreted [34]. Therefore, it is possible that Sda1 regulates FB_1_ biosynthesis in a similar way, where it controls the balance between *FUM* gene(s) expression and toxin production. The lack of growth of the *ace1* deletion strain in sorbitol is not understood. However, it has been suggested that Ace1 may act as a general transcriptional regulator, rather than a sequence-specific repressor of cellulase and xylanase expression [36]. TFs activate or repress gene expression by stabilizing or blocking the recruitment of the RNA polymerase complex by binding *cis* elements such as enhancers or repressors. Additionally, since TFs possess more than one binding domain, a single TF can have the capacity to act as an activator and a repressor [61], [62]. Therefore, it is reasonable to propose that Sda1 binds to different sets of *cis* elements depending on environmental cues to regulate carbon catabolism and secondary metabolism.

D-arabitol accumulation in Δsda1 was significantly decreased compared to wild type, when grown on maize kernels and in liquid media containing glucose. At least two arabitol metabolic pathways have been proposed for filamentous fungi based on substrate availability [63]–[70]. The first arabitol biosynthetic pathway proposed for fungi involves the conversion of L-arabinose to D-arabitol in five steps with four intermediate metabolites (L-arabitol, L-xylulose, xylitol, and D-xylulose in the order listed) [64], [67]–[70]. Additionally, conversion of D-xylose to xylitol has been proposed as a mechanism for synthesizing substrates for D-arabitol biosynthesis in *A. niger* [65], [70]. Together, arabinose and xylose account for over 70% of heteroxylan composition in maize bran [71], [72]. Therefore, the reduced accumulation of arabitol in Δsda1, on kernels, suggests that Sda1 is either involved in acquisition of arabinose and/or xylose from maize kernels or in regulating downstream metabolic processes. Given the associated role of *T. reesei* Ace1 in the negative regulation of cellulases and xylanases along with the increased growth of Δsda1 compared to the wild type on kernels, the role of Sda1 on arabitol accumulation is more likely to be in regulating metabolic steps downstream of arabinose and/or xylose rather than heteroxylan degredation or carbohydrate uptake [34].

The second arabitol biosynthetic pathway proposed for fungi utilizes glucose as a substrate for arabitol biosynthesis by conversion to either D-xylulose-5-phosphate (DX5P) or D-ribulose-5-phosphate (DR5P) [63], [64], [66]. DX5P and DR5P are then either dephosphorylated to their respective pentoses and reduced to D-arabitol [64], [68] or are reduced to D-arabitol-5-phosphate, which is dephosphorylated to D-arabitol [64], [66]. In *F. verticillioides*, it is not clear which pentose-phosphate intermediate is utilized or whether it is first dephosphorylated or first reduced. However, since Δsda1 accumulates less arabitol than the wild type when grown on glucose, it is apparent that, under the conditions tested in this study, Sda1 plays a role in the regulation of the pathway responsible for converting glucose to arabitol. While it is not clear which step in the pathway is regulated by Sda1, the accumulation of arabitol at reduced concentrations suggests that either Sda1 is not the sole regulatory factor or that more than one pathway for conversion of glucose to D-arabitol exist in *F. verticillioides*.

In addition to accumulating less mannitol when grown on glucose, Δsda1 exhibited significantly reduced growth on mannitol when compared to the wild type. In fungi, a cyclic pathway for mannitol metabolism was originally proposed [73], where fructose-6-phosphate is reduced to mannitol-1-phosphate (M1P) by mannitol phosphate dehydrogenase (MPDH). M1P is then dephosphorylated to mannitol, which is in turn oxidized to fructose by MtDH [4], [5], [64], [73]. However, knockout mutation of MPDH in both *Stagonospora nodorum* and *Alternaria alternata* resulted in reduction of mannitol accumulation to approximately 20% and 11.5% of wild type, respectively, when grown on glucose. This reduction was not seen in MPDH deletion mutants of *A. alternata* grown on fructose, which indicates that MtDH is capable of converting mannitol to fructose. Additionally, MPDH deletion mutants exhibited reduced growth on mannitol, indicating a function for MPDH in mannitol catabolism. While deletion of MtDH also reduced growth on mannitol in *S. nodorum*, this reduction in growth was not observed in *A. alternata* [4], [5]. The reduced growth on mannitol along with the reduction in mannitol accumulation in Δsda1 compared to wild type are consistent with a loss of function of MPDH as described for *S. nodorum* and *A. alternata*. Furthermore, the near wild-type level accumulation of mannitol in Δsda1 grown on kernels is consistent with direct conversion of available fructose to mannitol by MtDH. These data, along with the ability (albeit reduced) of Δsda1 to grow on mannitol and biosynthesize mannitol when grown on glucose, lead us to conclude that Sda1 is a positive regulator of MPDH, but is not involved in the regulation of MtDH under the conditions tested.

**Figure 9:**
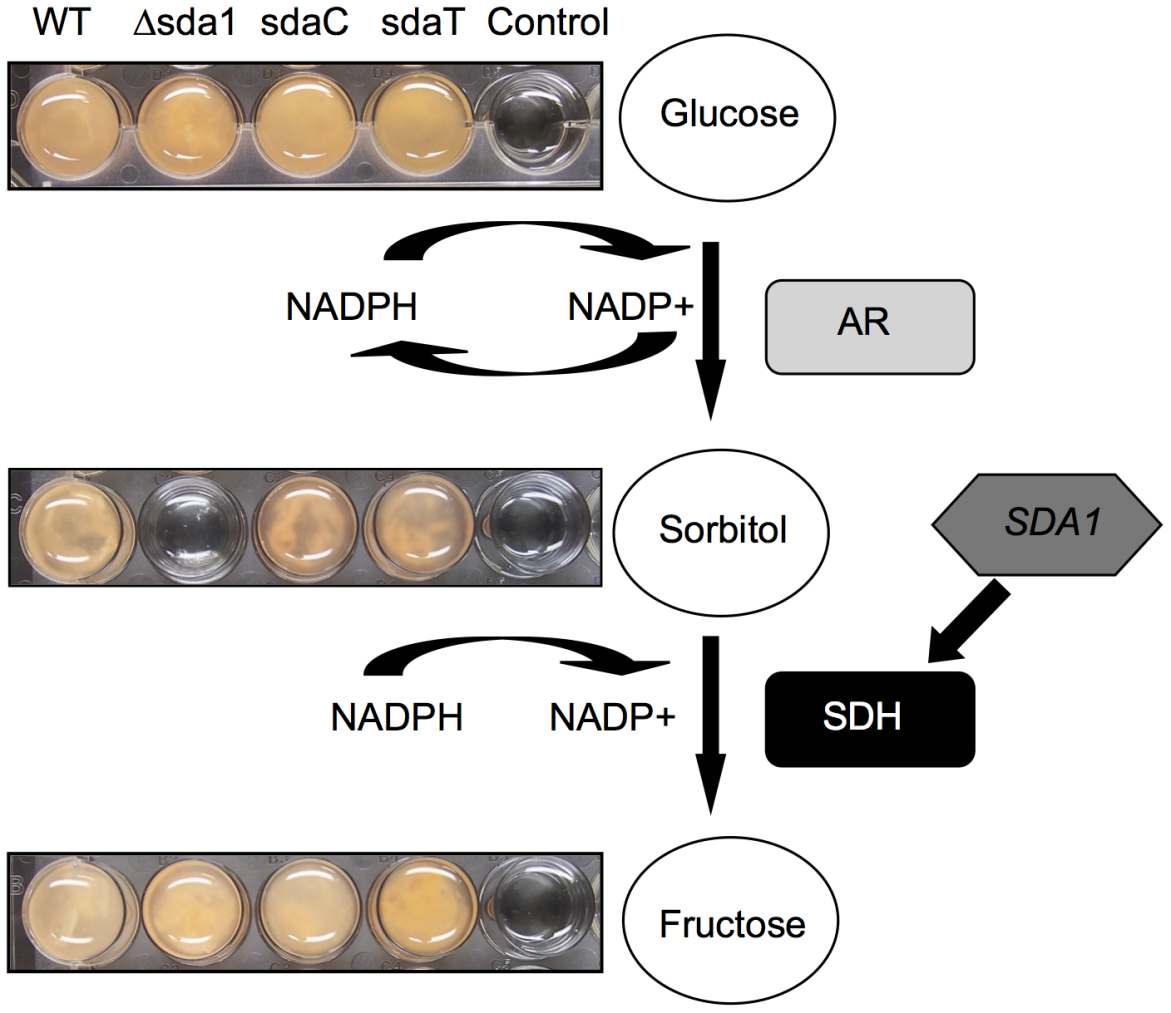
Proposed schematic model of Sda1 regulation in *F. verticillioides*. In mammals, the polyol pathway is composed by two enzymatic reactions, where glucose is reduced to sorbitol by aldose reductase (AR), and subsequently oxidized to fructose by sorbitol dehydrogenase (SDH). The model visualizes the putative transcriptional regulation of SDH by Sda1 in *F. verticillioides*.

In conclusion, we postulate that in the presence of sorbitol Sda1 transcriptionally activates SDH gene expression via the polyol pathway (Figure 9). In mammals, the polyol pathway has been studied in detail due to the fact that a deficiency in this pathway has been linked to vascular and neurological complications of diabetes [74]. The polyol pathway is composed of two enzymatic reactions, in which glucose is reduced to sorbitol by aldose reductase (AR), and subsequently oxidized to fructose by SDH. In the presence of glucose, AR utilizes NADPH as a co-factor to reduce the aldehyde group to sorbitol [75]. Significantly, Brahmachari et al [76] suggested that AR up-regulation is due to the presence of *cis*-elements in its promoter region, which may play a role on chromatin rearrangements or the enhancement of recruitment of TF complexes. SDH is a member of the medium chain dehydrogenase/reductase super family that uses NAD(H) as a co-factor. Significantly, in this study we observed an increase of expression of *SDH1,* a putative human *SDH* ortholog, in the wild type when grown on sorbitol medium. However, further studies are needed in order to corroborate the existence of a fungal SDH ortholog and how polyol metabolism influences fungal physiology.

## Supporting Information

Figure S1 **Amino acid alignment of** ***F. verticillioides*** **Sda1 and** ***T. reesei*** **Ace1 using ClustalW program.** Sda1 shares 65% identity and 76% similarity with Ace1. The conserved residues were black shaded and boxes indicate the regions corresponding to the three zinc fingers. Asterisks indicate the zinc coordinating Cys and His residues, and an arrow shows the first methionine shown in yeast to be sufficient for activation [45].
(TIFF)

Figure S2 **Quantification of ergosterol contents in** ***F. verticillioides*** **strains when grown in non-viable autoclaved maize kernels.** Wild-type (WT), Δsda1, sdaC, and sdaT strains were point inoculated with an agar plug (0.5 cm in diameter) on sterile corn kernels and incubated for 14 days at 25°C under a 14-h light/10-h dark cycle. Ergosterol contents (ppm) were quantified by high-performance liquid chromatography (HPLC) analysis. All values represent the means of three biological replications with standard errors shown as error bars, and two independent experiments showing similar results.
(TIFF)

Figure S3 **Growth comparison of wild-type (WT) and** Δ**sda1 strains on DL agar plates amended with 2% sorbitol.** Strains were grown for 7 days at 25°C. Note that with the addition of agar there is no growth difference between the strains.
(TIFF)

Figure S4 **Biomass quantification of** ***F. verticillioides*** **strains.** Wild-type (WT) and Δsda1 strains were grown on DL media amended with 2% of cellulose and xylan. DL media with 2% of glucose and xylose were used as controls. The mycelia of each strain were harvested after 6 days of incubation in DL medium and dried at 100°C for 24 hrs. Results are the means of three and four biological replications with standard errors shown as error bars, and repeated at least twice.
(TIFF)

Figure S5 **HPLC chromatograms.** Fungal strains were grown for 7 days on (A) maize kernels and (B) in liquid media containing glucose as the carbon source. (A) Peak: 1, arabitol; 2, mannitol; 3, sorbitol; 4, internal standard; 5, trehalose. (B) Peak: 1, arabitol; 2, mannitol; 3, internal standard; 4, trehalose.
(TIFF)

Table S1 List of primers used in this study.
(DOCX)

Table S2 Comparison of *SDA1* expression levels in *F. verticillioides* wild type versus sdaC when utilizing glucose or sorbitol as the sole source of carbon.
(DOCX)
