# Supplementary figures and images for: Sda1, a Cys2-His2 Zinc Finger Transcription Factor, Is Involved in Polyol Metabolism and Fumonisin B1 Production in Fusarium verticillioides
Source: PLoS One. 2013 Jul 3;8(7):e67656. doi: 10.1371/journal.pone.0067656 (PMC3700993; doi:10.1371/journal.pone.0067656)

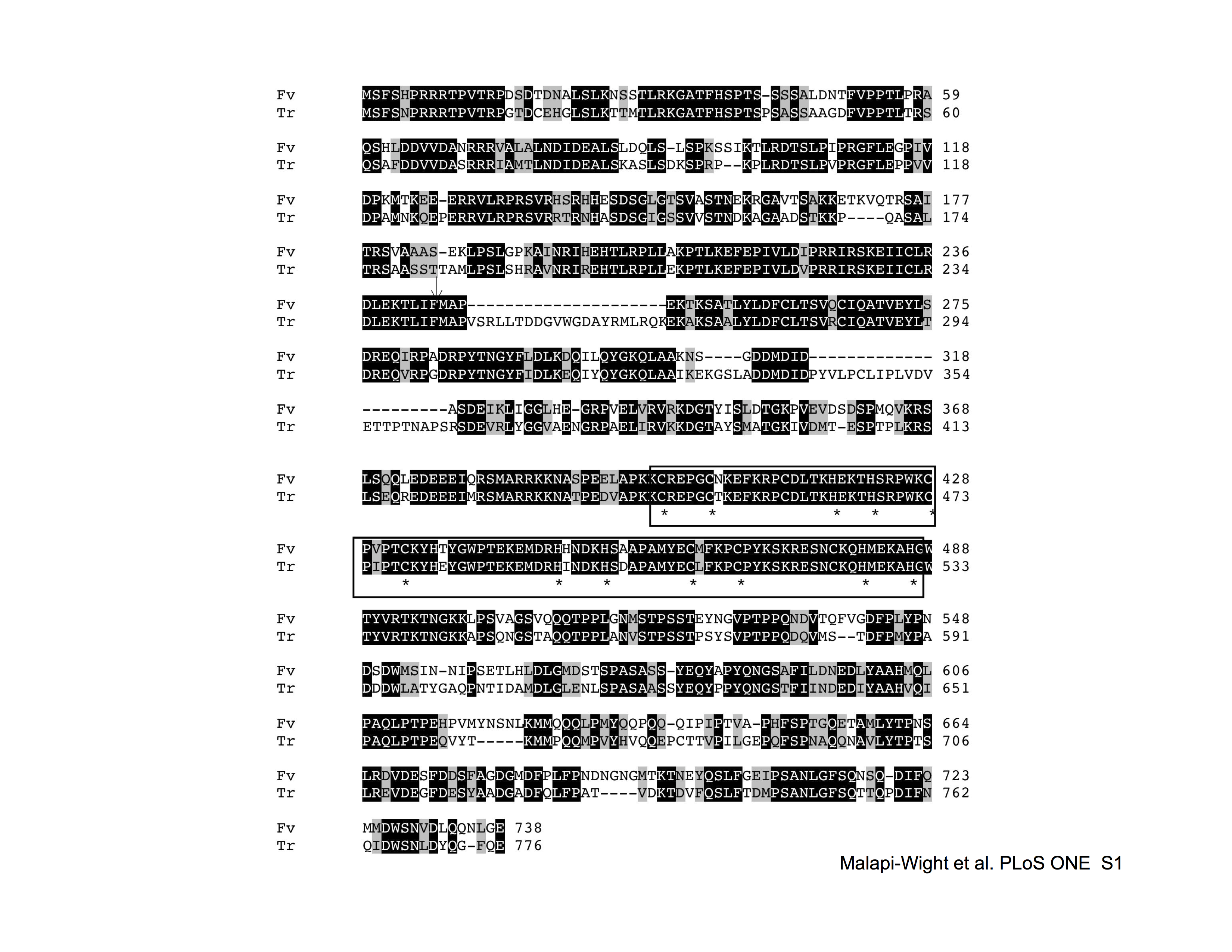

Supplement: Figure S1 — Amino acid alignment of F. verticillioides Sda1 and T. reesei Ace1 using ClustalW program. Sda1 shares 65% identity and 76% similarity with Ace1. The conserved residues were black shaded and boxes indicate the regions corresponding to the three zinc fingers. Asterisks indicate the zinc coordinating Cys and His residues, and an arrow shows the first methionine shown in yeast to be sufficient for activation [45]. (TIFF) [file pone.0067656.s001.tif]

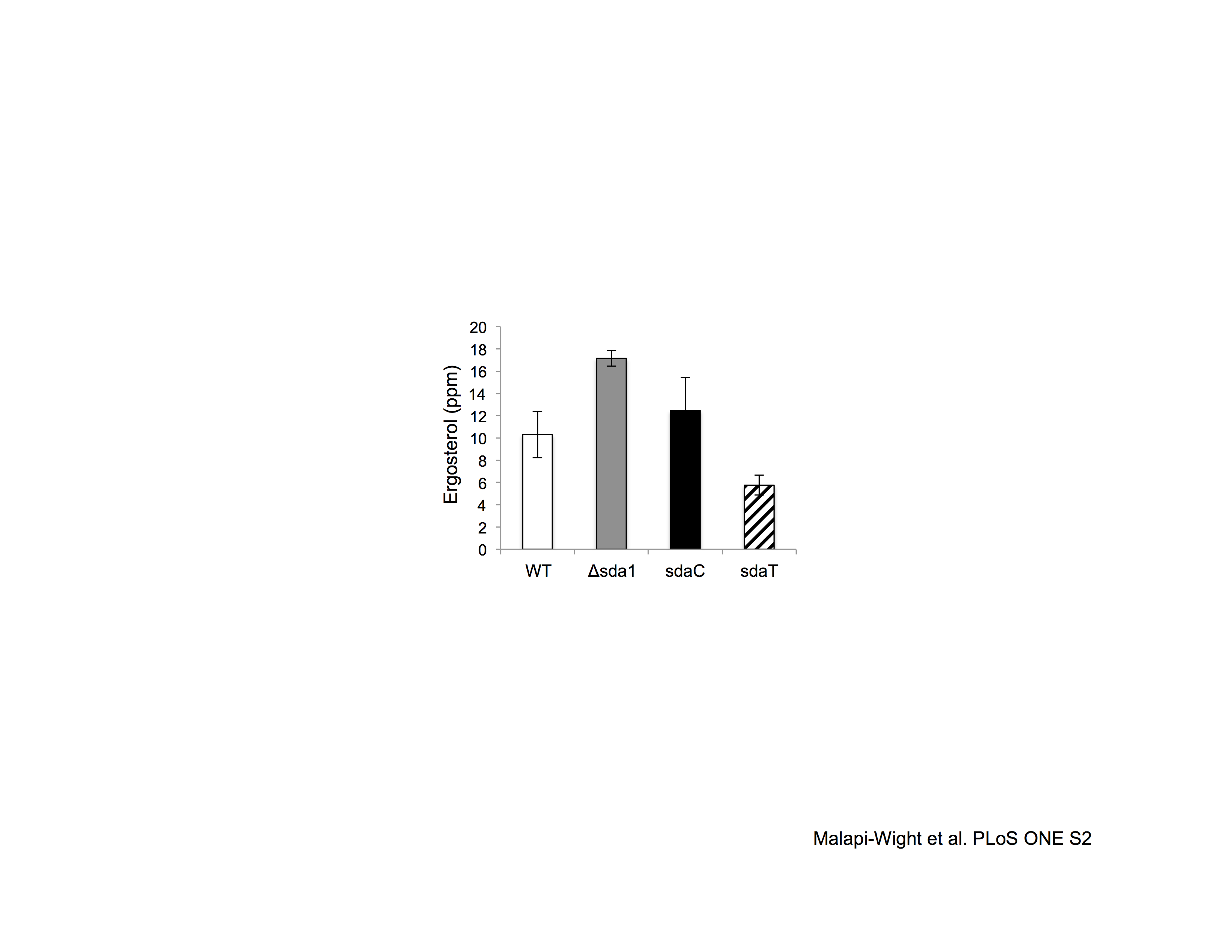

Supplement: Figure S2 — Quantification of ergosterol contents in F. verticillioides strains when grown in non-viable autoclaved maize kernels. Wild-type (WT), Δsda1, sdaC, and sdaT strains were point inoculated with an agar plug (0.5 cm in diameter) on sterile corn kernels and incubated for 14 days at 25°C under a 14-h light/10-h dark cycle. Ergosterol contents (ppm) were quantified by high-performance liquid chromatography (HPLC) analysis. All values represent the means of three biological replications with standard errors shown as error bars, and two independent experiments showing similar results. (TIFF) [file pone.0067656.s002.tif]

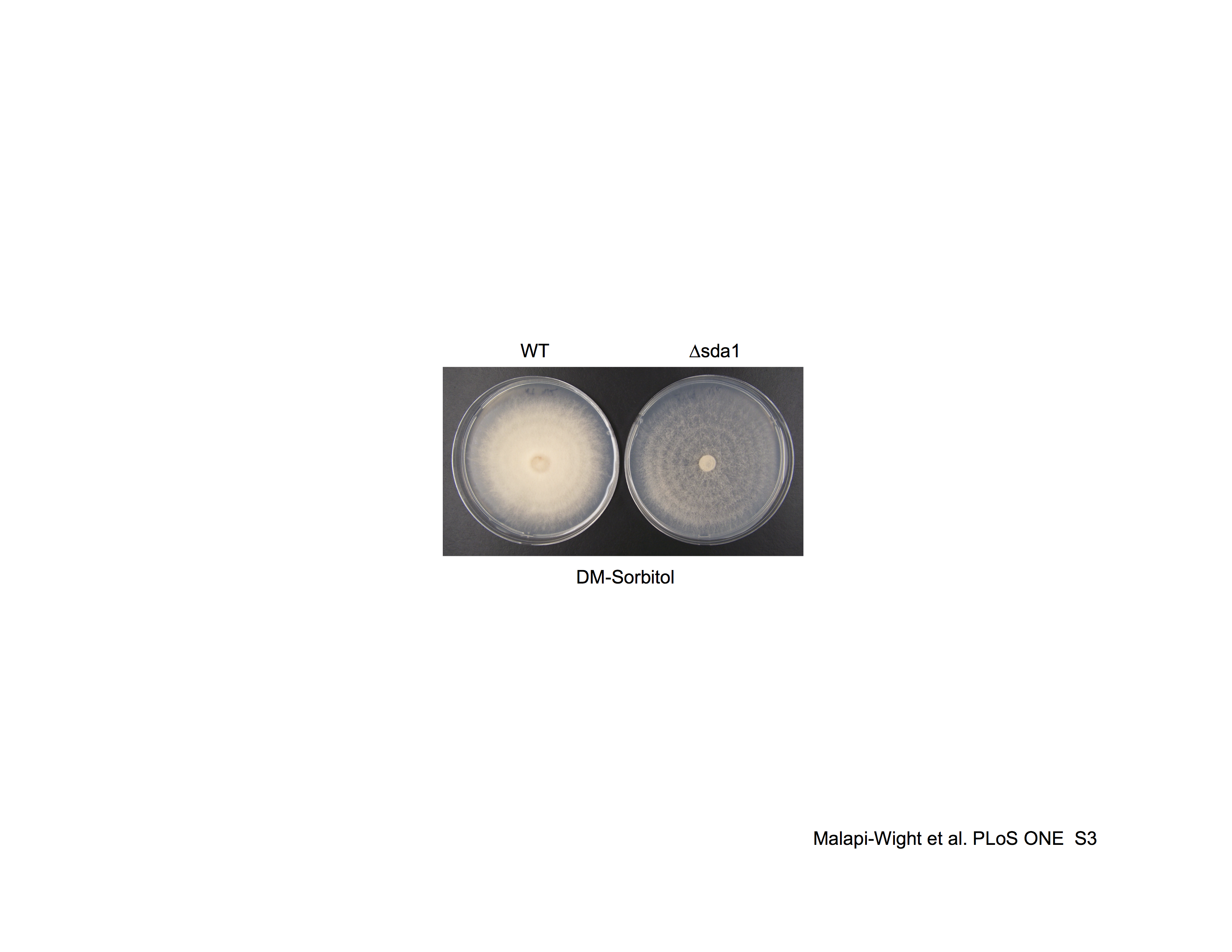

Supplement: Figure S3 — Growth comparison of wild-type (WT) and Δsda1 strains on DL agar plates amended with 2% sorbitol. Strains were grown for 7 days at 25°C. Note that with the addition of agar there is no growth difference between the strains. (TIFF) [file pone.0067656.s003.tif]

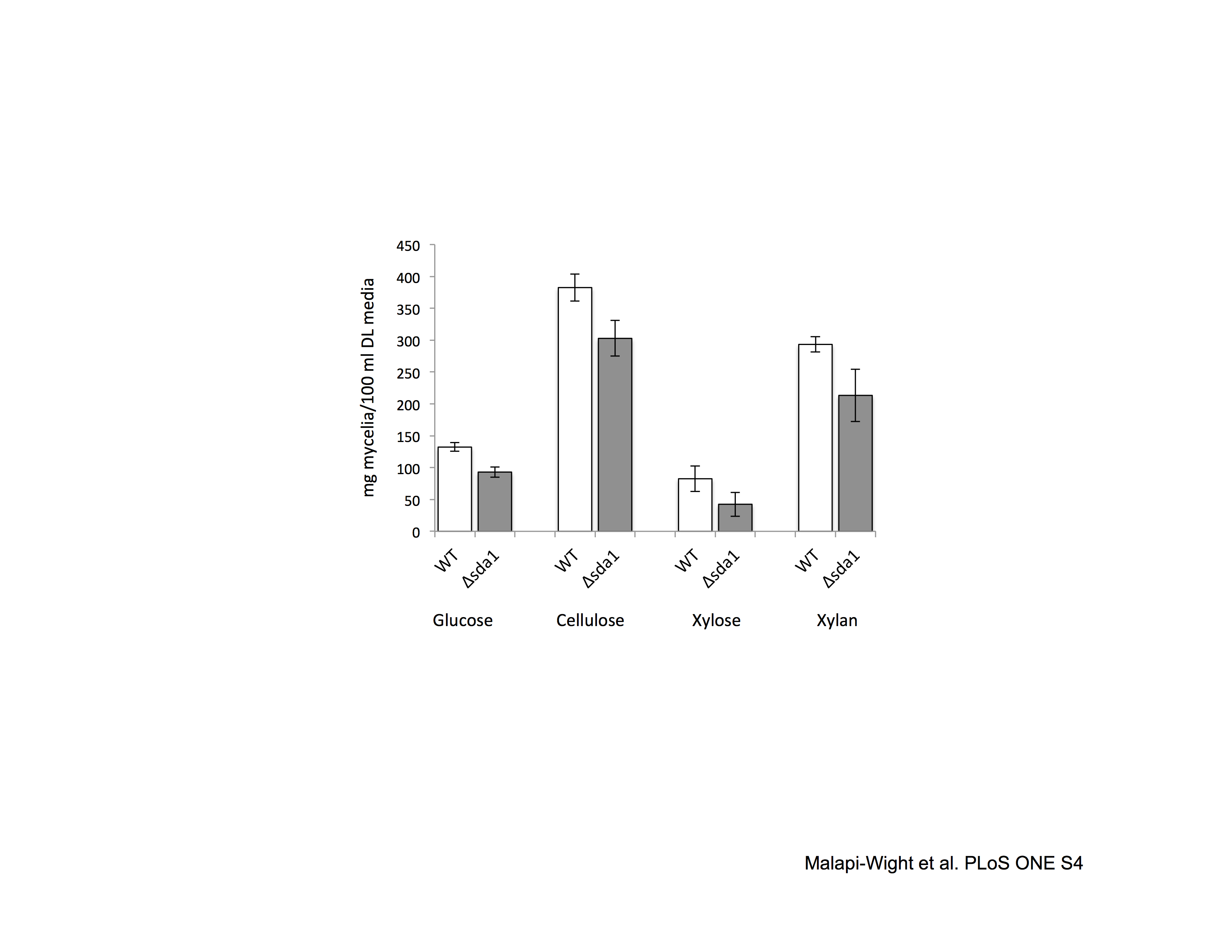

Supplement: Figure S4 — Biomass quantification of F. verticillioides strains. Wild-type (WT) and Δsda1 strains were grown on DL media amended with 2% of cellulose and xylan. DL media with 2% of glucose and xylose were used as controls. The mycelia of each strain were harvested after 6 days of incubation in DL medium and dried at 100°C for 24 hrs. Results are the means of three and four biological replications with standard errors shown as error bars, and repeated at least twice. (TIFF) [file pone.0067656.s004.tif]

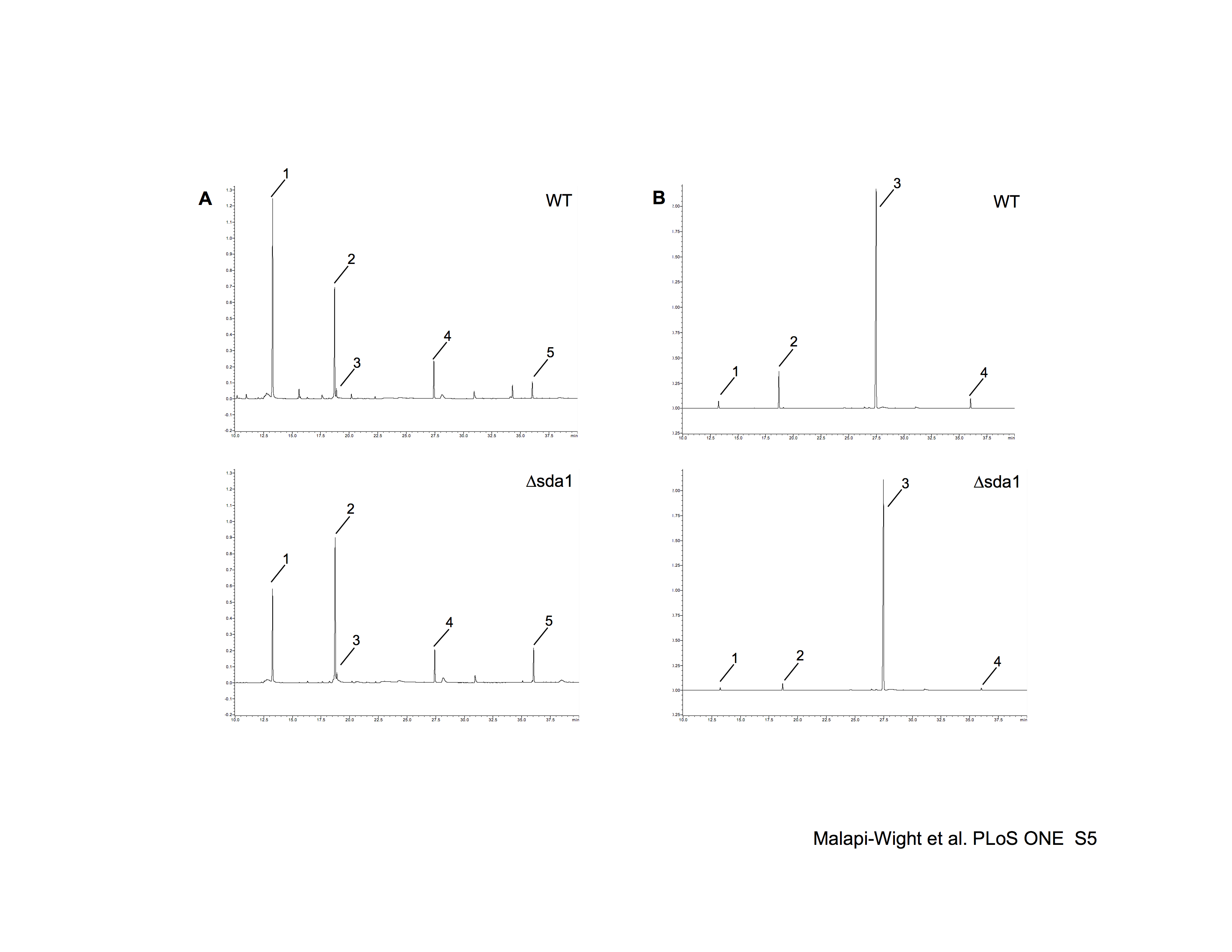

Supplement: Figure S5 — HPLC chromatograms. Fungal strains were grown for 7 days on (A) maize kernels and (B) in liquid media containing glucose as the carbon source. (A) Peak: 1, arabitol; 2, mannitol; 3, sorbitol; 4, internal standard; 5, trehalose. (B) Peak: 1, arabitol; 2, mannitol; 3, internal standard; 4, trehalose. (TIFF) [file pone.0067656.s005.tif]
