# Supplementary material for: Sda1, a Cys2-His2 Zinc Finger Transcription Factor, Is Involved in Polyol Metabolism and Fumonisin B1 Production in Fusarium verticillioides
Source: PLoS One. 2013 Jul 3;8(7):e67656. doi: 10.1371/journal.pone.0067656 (PMC3700993; doi:10.1371/journal.pone.0067656)
Supplement: Table S1 — List of primers used in this study. (DOCX) [file pone.0067656.s006.docx]

**Table S1.** List of primers used in this study

| **Name** | **Sequence (5’→3’)** |
| --- | --- |
| TF9-LF-F | TTGCAATGTGATCACCTCGC |
| TF9-LF-R | TAGATGCCGACCGGGAACATAGGGATCCAGTCTTCTGC |
| TF9-RF-F | CCACTAGCTCCAGCCAA GATAAAAGAGACAGTCGACGG |
| TF9-RF-R | GGAATTGAGGTGAGTCTTGGTG |
| Ace1-BamH-F | GCAGGATCCTGCGAGATCTCGAGAAGACC |
| Tr-Ace1-R | TGGGTTCGATTCCCAGTATCG |
| Tr-Ace1-NesR | AGTATCGGAGAACATCGTCTTCACC |
| GPDA-F-Hyg-tail | CATGGTCATAGCTGTTTCCTGAGAGACGGACGGACGCAGAG |
| GPDA-F-Hyg | AGCTTGTATCTCTACACACAGG |
| GPD-BamH-R | CGCGGATCCAGAAAAGAAAAGAGCAGCTGG |
| HYG/F | CTTGGCTGGAGCTAGTGGAGGTCAA |
| HY/R | GTATTGACCGATTCCTTGCGGTCCGAA |
| YG/F | GATGTAGGAGGGCGTGGATATGTCCT |
| HYG/R | GTTCCCGGTCGGCATCTACTCTAT |
| SDA1+qF | GCCTGCTTCGGCTAGTTCCT |
| SDA1+qR | GGCCGAGCCATTCTGGTAT |
| XDH1-q-F | ACAGCATCTCCCCCGGATA |
| XDH1-q-R | CCTGGGTCTCAGCGTCGATA |
| SDH1-q-F | ACTGCGCCTGCGGATTT |
| SDH1-q-R | CACCCTCCTGCTGCGAAA |
| SDH2-q-F | CTGCAAGTCCGGACGATACA |
| SDH2-q-R | GGGATCCGCAGCGAACT |
