# Supplementary material for: Sda1, a Cys2-His2 Zinc Finger Transcription Factor, Is Involved in Polyol Metabolism and Fumonisin B1 Production in Fusarium verticillioides
Source: PLoS One. 2013 Jul 3;8(7):e67656. doi: 10.1371/journal.pone.0067656 (PMC3700993; doi:10.1371/journal.pone.0067656)
Supplement: Table S2 — Comparison of SDA1 expression levels in F. verticillioides wild type versus sdaC when utilizing glucose or sorbitol as the sole source of carbon. (DOCX) [file pone.0067656.s007.docx]

**Table S2.** Comparison of *SDA1* expression levels in *F. verticillioides* wild type versus sdaC when utilizing glucose or sorbitol as the sole source of carbon ^a, c^

|  | **WT ^b^** | **sdaC ^b^** |
| --- | --- | --- |
| *SDA1* expression in glucose | 1± 0.79 | 4.20 ± 0.51 |
| *SDA1* expression in sorbitol | 1± 0.65 | 2.58 ± 0.39 |

^a^ Total RNA samples were prepared from *F. verticillioides* wild-type and sdaC strains grown on DL media + 2% w/v glucose or sorbitol. Mycelia were collected after 70 hours post inoculation. Real time quantitative reverse transcription (qRT)-PCR analysis of gene expression was performed with SYBR-Green as the fluorescent reporter. Gene expression was normalized to endogenous β-tubulin gene expression.

^b^ The gene expression was calibrated using 2^-ΔΔCt^ method. Data represent the relative expression, where *SDA1* gene expression in the wild-type strain in standardized to 1.00 ± the standard error of dCT values (*n=3*).

^c^ Each value is the mean of 3 technical replicates from one biological experiment. A biological replication was performed with no statistically different results.
